# pblat: a multithread blat algorithm speeding up aligning sequences to genomes

**DOI:** 10.1186/s12859-019-2597-8

**Published:** 2019-01-15

**Authors:** Meng Wang, Lei Kong

**Affiliations:** Center for Bioinformatics, State Key Laboratory of Protein and Plant Gene Research, School of Life Sciences, Peking University, Beijing, 100871 People’s Republic of China

**Keywords:** Sequence alignment, Genome annotation, Parallel computing, Cluster computing

## Abstract

**Background:**

The blat is a widely used sequence alignment tool. It is especially useful for aligning long sequences and gapped mapping, which cannot be performed properly by other fast sequence mappers designed for short reads. However, the blat tool is single threaded and when used to map whole genome or whole transcriptome sequences to reference genomes this program can take days to finish, making it unsuitable for large scale sequencing projects and iterative analysis. Here, we present pblat (parallel blat), a parallelized blat algorithm with multithread and cluster computing support, which functions to rapidly fine map large scale DNA/RNA sequences against genomes.

**Results:**

The pblat algorithm takes advantage of modern multicore processors and significantly reduces the run time with the number of threads used. pblat utilizes almost equal amount of memory as when running blat. The results generated by pblat are identical with those generated by blat. The pblat tool is easy to install and can run on Linux and Mac OS systems. In addition, we provide a cluster version of pblat (pblat-cluster) running on computing clusters with MPI support.

**Conclusion:**

pblat is open source and free available for non-commercial users. It is easy to install and easy to use. pblat and pblat-cluster would facilitate the high-throughput mapping of large scale genomic and transcript sequences to reference genomes with both high speed and high precision.

## Background

Blat [1] is a sequence alignment tool designed to map DNA, RNA and protein sequences to reference genomes. It is commonly used to locate sequences in a reference genome, find homologous sequences from genomes of closely related species, identify exon-intron boundaries from mRNA sequences and determine gene structures, and to help assemble and annotate genome and transcriptome sequences [2]. Although many fast sequence aligners, like BWA [3] and Bowtie [4], have been developed to map short sequence reads generated by high-throughput sequencing, they are not capable of mapping long reads or sequences with abundant gaps or spliced sequences from discontinues genomic regions [5]. In contrast, blat is an ideal tool for such applications with its high sensitivity and precision [6, 7].

However, with the increasing quantity of sequences generated by high throughput sequencing projects, blat cannot meet the speed requirements needed for large-scale analysis and regularly updated annotations. For example, when used to map the whole transcriptome sequences of vertebrates to a reference genome, it would take days to finish using blat. This is due to the blat algorithm being single threaded and, thus, not taking full advantage of modern multicore processors. One might use the GNU parallel [8] tool to execute multiple instances of blat in parallel using one or more computers. However, each blat process would load a copy of the whole reference genome and build and store the index of the genome into memory, which might exceed the available physical memory of conventional computers if multiple blat processes run simultaneously.

To overcome these limitations, we present pblat (parallel blat), which functions to speed up blat alignments by implementing multiple thread and cluster computing support. With pblat, all threads share the same memory copy of the whole reference genome and the index. As such, pblat utilizes almost the same amount of memory as blat. The run time is reduced with the number of threads used, and the output results of pblat are identical with that of blat. The cluster version of pblat (pblat-cluster) runs on computer clusters with MPI (Message Passing Interface) support, which is able to help reduce the run time of blat from days to minutes.

## Implementation

pblat extends the blat algorithm with multiple thread support by employing POSIX threads via the C programming language. pblat employs data-level parallelism. The input query file in FASTA format is virtually divided into the same number of parts as the number of threads specified using the ‘-threads’ command line option. Each part is comprised of the same number of query sequences to load balance among threads. Each thread takes one part of the input sequences and performs the blat algorithm to align the sequences to the genome. Only one copy of the genome sequences is loaded into memory and all the threads share this memory to query the input sequences against the genome. This makes the memory consumption of pblat keep to the minimum no matter how many threads are used. The outputs of each thread are written to an independent temporary file. After all the threads finish, all of the temporary output files are combined to form the final output file. This ensures that the order of output records corresponds to the order of query sequences in the input file no matter how many threads are used. All of the global variables and static variables in the original blat program are localized to ensure all the variables and subroutines are thread safe.

The cluster version of pblat extends the multithread version of pblat with MPI support. The master process of the pblat-cluster distributes the virtual input query file parts to all of the spawned processes in each computing node via MPI. The spawned processes running in the same computing node are automatically merged and switched to the multithread mode of pblat, sharing the same memory copy of the reference genome and index to minimize the memory requirement per computing node. After all the spawned processes in each computing node finish, the master process combines all of the output files generated by each process to form the final output file.

## Results

### Performance evaluation of pbalt

We evaluated the performance of pblat using different number of threads and compared to the results of the original blat. All analyses were performed on a Linux machine with 4 Intel Xeon E7–4830 @ 2.13GHz CPUs (32 cores in total, 2 threads per core) and 128G memory. We employed the nucleotide sequences of all human protein-coding transcripts with length range from 1 kb to 5 kb in the GENCODE release 26 [9]. We aligned these sequences to the GRCh38.p10 human reference genome sequences with blat and pblat. The test data consisted of 42,286 transcript sequences. The mean length of the test transcripts was 2440 and the median length was 2226. The blat and pblat analyses, with 2, 4, 8, 16, 24, 32, 40, 48, 56 and 64 threads, were executed to map all the transcripts to the genome.

**Fig. 1:**
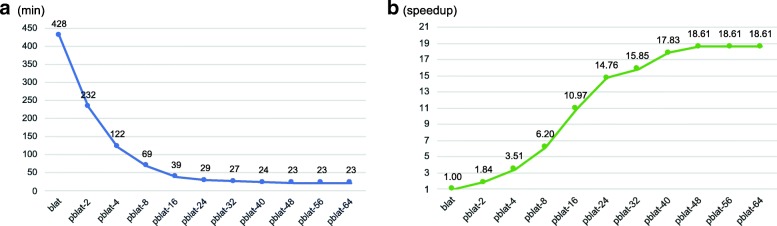
Performance evaluation of pblat. **a** timing benchmarks of blat and pblat using different thread numbers (from 2 to 64). Each time represents the mean of three independent executions performed with the same arguments and on the same machine. **b** Speedup of pblat with different number of threads, compared to blat

The results showed that pblat reduced the run time relative to the increasing number of threads used (Fig. 1a). Blat took more than 7 h to map all the test transcripts to the reference genome, whereas pblat with 16 threads only required approximately 39 min to finish, which was 10.97x speedup than the blat. From 2 threads to 32 threads, the speedup of pblat increased with the increasing number of threads used (Fig. 1b). When using 2 to 8 threads, the speedup increased almost linearly with the number of threads. But the speed of acceleration decreases when using more threads and the run time did not further reduce after 32 threads. The final speedup of this test was 18.61 when using 48 threads. Due to the channel and bandwidth limitation of memory accessing, the acceleration was not proportional to the number of threads used. The memory usage for pblat with any number of threads tested was almost the same as that of blat. The results generated by pblat were completely identical to the results of the blat analysis. Based on these results, we suggest to use half of total CPU cores to get the maximum acceleration, or setting the number of threads as the total number of memory channels to get a high acceleration with economic CPU resource consumptions.

To evaluate the performance of pblat on real long-read sequencing data with sequencing errors leading to abundant mismatches and gaps, we adopted the mouse single-cell full length transcript RNA-seq data sequenced on the long-read single-molecule Oxford Nanopore MinION sequencer (ONT) [10]. The dataset was downloaded from NCBI Sequence Read Archive (SRA) with accession number SRR5286960. For a single murine B1a cell, 104,990 reads were generated by ONT with the maximum length 6296 bp, mean length 1868 bp and median length 1787 bp. Fast spliced read aligners including HISAT2 [11], STAR [12] etc., were not compatible with such long reads with high error rates [10]. In the original study [10], the blat was employed to align these nanopore long reads to the mouse genome and successfully helped identify novel transcript isoforms. We replicated the aligning step using blat and pblat with 8 and 16 threads. The blat program took 935 min to align all these reads to GRCm38 genome (primary assembly), while pblat with 8 threads used 149 min and pblat with 16 threads took 86 min to finish. The speedup for pblat with 8 and 16 threads relative to blat was 6.28 and 10.87, respectively. The speedup was consistent with results in the last analysis. These results showed pblat could significantly accelerate aligning long sequencing reads generated by the Oxford Nanopore and PacBio SMRT (Single Molecule Real-Time) sequencing platforms.

### Performance evaluation of pblat-cluster

**Fig. 2:**
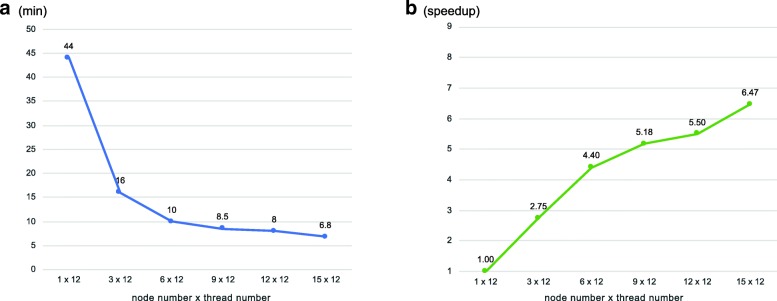
Performance evaluation of pblat-cluster. **a** timing benchmarks of pblat-cluster using different number of computing nodes (from 1 to 15), with 12 threads per node. Each time represents the mean of three independent executions performed with the same arguments and on the same cluster. **b** Speedup of pblat-cluster with different number of computing nodes, compared to pblat with one node 12 threads

The performance of pblat-cluster was evaluated on a high-performance computing cluster with 15 nodes. Each node had 24 CPU cores @ 2.30GHz (1 thread per core) and 128GB memory. All the nodes were connected by the InfiniBand network and shared a GPFS file system. All the nodes ran Linux system with OpenMPI 3.0.0. The test data was the same as that used in the evaluation of pblat. We evaluated the pblat-cluster with 1, 3, 6, 9, 12 and 15 computing nodes. Each node ran 12 threads. Results indicated that the run time decreased significantly with the increasing number of computing nodes employed (Fig. 2). The blat program took 6.4 h with one thread on one node to align all the test sequences to the reference genome. The pblat-cluster with one node (12 threads) used 44 min. When using 15 nodes, pblat-cluster reduced the time consumption to 6.8 min, which was 6.47x speedup than pblat with 12 threads in one node and 51.18x speedup than blat. The speedup would further increase with more computing nodes, but as pblat the acceleration would not be proportional to the number of nodes employed and there is a roof for the maximum speedup. The run time of pblat-cluster is determined by the slowest node. When a node has an extremely long sequence to align which takes much longer time than the other sequences, the total time consumption would be determined by the time used to align this sequence no matter how many nodes are used.

We then compared the time consumption when running pblat with 12 threads and that when running pblat-cluster with 12 nodes (1 thread per node). The pblat with 12 threads took 44 min to align all the test transcripts to the reference genome. The pblat-cluster with 12 nodes used 39.8 min. As expected, pblat-cluster is faster than pblat using the same number of threads, because for pblat-cluster the threads run on different computing nodes, with each node using its own memory of genomes and indexes, they do not compete to access the memory.

## Conclusion

pblat is open source and the source code is easy to compile by simply typing ‘make’ to generate the binary program on Linux or Mac OS systems. pblat is also easy to use, and all the command-line options are the same as blat, with only the addition of the ‘-threads’ option to specify the number of threads to use. Pipelines employing blat could directly switch to using pblat. pblat enables users to take advantage of both the high precision of blat and the high speed available with other popular sequence aligners. Overall, pblat facilitates the rapid analysis and annotation of whole genome and whole transcriptome sequences.

## Availability and requirements

**Project name:** pblat

**Project home page:** http://icebert.github.io/pblat, http://icebert.github.io/pblat-cluster

**Operating systems:** Linux, Mac OS X

**Programming language:** C

**License:** The source code and executables are freely available for academic, nonprofit and personal use. Commercial licensing information is available on the Kent Informatics website (http://www.kentinformatics.com).

**Any restrictions to use by non-academics:** license needed

## Abbreviations

GPFS: General parallel file system
MPI: Message passing interface
